# A Dialogue between a Bilious Patient and a Physician

**Published:** 1839-04

**Authors:** 


### ART. VIII.

*A Dialogue between a Bilious Patient and a Physician.* By JAMES HENRY, M.D., Fellow of the College of Physicians, Dublin. *Third Edition.—Dublin,* 1838. 8vo. pp. 52.

THIS is one of the most sensible productions we have met with for a long time, and is, in our opinion, calculated to do much good, not only to the innumerable lay dabblers in physic, who come within the class of "bilious," but to the grave professors of legitimate medicine themselves. It is written in an accurate and pure style, and the whole of the argument is so clearly placed before the reader, and is so logically and forcibly reasoned throughout, that it cannot fail to produce a decided effect on the mind of every sensible and unprejudiced man. The great object which the author has in view is "to show that the habitual use of purgatives is injurious to health, and that the diseases commonly denominated nervous and bilious cannot be cured by these medicines, nor indeed by any medicines whatever, but solely by avoiding the causes from which those diseases spring:" and it would appear from his preface that, on this account, he has been accused of a desire to depreciate medicines and the art of medicine. We, however, entirely acquit him from such a charge; being thoroughly convinced that the true interests of the medical profession can never suffer from enlightened attempts to instruct the public on the subject of their health, and feeling, with the author, assured "that, in attempting to correct, as far as in his power, a great medical abuse, he is doing that which not only will not diminish, but on the contrary will increase and promote, a rational confidence in the healing art." It is not to writers like Dr. Henry, who come openly forward to disseminate the great truths of physic, without any self-interested motive, that the guardians of professional honour will ever seek to apply the lash, but to those base and dishonest men who, under the thin pretence of communicating information to the public, plot in reality how they may lure them into their toils.

In the first part of the dialogue, a vivid picture is drawn of the misery of a bilious or dyspeptic and nervous patient, and of the flattering but delusive and merely temporary relief afforded by purgatives; a picture, of which every physician will admit the truth, who has had much experience in such cases, and who knows the ordinary mode of treatment of them by routine practitioners or the patients themselves. After a complete exposure of the inadequacy and injurious effects of the purging practice, the author—in reply to his patient's objection that "the necessity for a purgative is sometimes so pressing that present relief must be sought for, regardless of future consequences;" and that "the sentence of condemnation cannot be pronounced against those mildly opening and alterative medicines which neither sicken nor gripe, but merely give a gentle stimulus to the bowels, and assist their natural action;"—enters into an elaborate discussion to show that the established creed respecting the necessity of having a daily evacuation of a certain consistence and colour is utterly false: it thus concludes:

"I have now shown you that your opinion, that it is necessary to good health that the stools should be brought, by the aid of purgatives, to agree with a certain assumed standard, either with respect to frequency, or colour, or consistence, is opposed, 1st, by your own experience of the utter futility of all attempts to render your bowels more regular by the aid of purgative medicines. 2dly. By my medical experience of the same fact with respect to other persons. 3dly. By my medical experience that such attempts are not only futile but ruinous to the health. 4thly. By my medical experience of the great variety that obtains in the stools of healthy persons, both with respect to their number, colour, and consistence; or, if I may so say, by my medical experience of the non-existence of a fixed standard for the stools. 5thly. By the principles of physiology, which show that the stools of healthy persons must of necessity vary, and cannot by possibility be reduced to a fixed standard." (p. 27.)

He accounts for the great popularity, or, we may indeed say, of the nearly universal use of purgative medicines, by the great immediate relief afforded by them, by the real or supposed facilities they furnish for the indulgence in luxurious habits, by the fear that constipation, if unchecked, may lead to dangerous results, by the influence of traditionary opinion, by the misapprehension of the advice of Locke given in his Essay on Education, and by the direct and most extended authority of the late Mr. Abernethy, (the author might have added Dr. Hamilton,) and by the great recommendation of the practice to British practitioners from its simplicity and facility, and its harmonizing so happily with the empirical and *effective* practice so long characteristic of medical practice in this country; and, finally, by the efforts of the host of pill-makers, called into existence by these potent and multifarious causes on the infallible principle of statistical economy that supply will follow demand. These last two causes are happily explained in the following passage:

"Under this system a careful and laborious investigation into the particular circumstances of each case was no longer necessary: well prepared with his purgatives, the physician was ready for every case which might occur. If he understood the case, he gave purgatives, because he was convinced that they were required; if he did not understand the case, or had not leisure, or inclination, or ability to investigate it, he still gave purgatives; and thus, if he did not cure, he at least purged the patient, and so avoided the appearance of not knowing what should be done, and of standing an idle and inactive spectator of the progress of the disease. Thus were purgatives at one and the same time the offensive and defensive armour of the physician; the keen weapons with which he combated all diseases, and the secure coat of mail which covered his own ignorance, incapacity, or inattention. The practice of physicians to prescribe and recommend opening medicines, and the general use made of them by the public, produced of course a great demand for medicines of this class: the manufacture and sale of purgatives therefore became a profitable employment, and was carried on extensively by great numbers of persons, who, deriving large incomes from the sale of their medicines, took infinite pains, by means of advertisements in all the newspapers and periodicals, and by agents in almost every town in the empire, to give notoriety and celebrity to their nostrums, and at the same time to keep up the credit of the purging system, on which the sale of their drugs, and of course their revenues, entirely depended." (p. 35.)

Having convinced his patient of the groundlessness of the system of curing bilious complaints by purgatives, and of the utter inadequacy of these means to accomplish the end desired, the physician enters upon the exposition of his own *rational* system, which has the great merit of containing nothing that is novel, and nothing but what every experienced physician, who is accustomed to think and reason as well as to observe and prescribe, will admit the truth of. This system comprehends merely due attention to air, exercise, and diet.

"Your symptoms having arisen from the inability of your stomach properly to digest your food, and air and exercise affording the natural, and certain, and well-known means of strengthening the stomach and increasing its digestive power, it follows that if your stomach is not (as I believe it is not) already injured beyond recovery, and if you are careful not to injure it in future, you have only to take sufficient exercise in the open air, in order to render your stomach equal to the digestion of your food, and so obtain the perfect recovery of your health. You have for many years lived an anxious, sedentary life; you have passed much of your time in close, badly-ventilated apartments, and have taken but little exercise or healthful recreation: you must change your habits in all these particulars; you must give less time to business and sedentary occupations, and more time to exercise and recreation in the open air. If your circumstances do not permit you to ride, or hunt, or shoot, or course, you can at least afford some time for quick walking: if the middle of the day is engaged, you can rise early and walk before breakfast; or, if that time also is devoted to business, you can take an hour's walk at night, just before bedtime; a practice quite free from danger to those who have not delicate lungs, and which has the advantage of warming the skin, and particularly the feet, before going to bed, and of composing and refreshing the mind after the fatigues and business of the day. . . If you are too timid to go out at night in bad weather, you can practise dancing, or fencing, or sparring, or some other gymnastic exercise at home; or you can play with young people or children at some of their cheerful games; or you can read for an hour in a loud voice, an exercise celebrated even among the Romans for the cure of bilious diseases, but most unaccountably neglected in modern times, although it has not only the effect of strengthening the stomach and assisting the action of the bowels, but also of bracing the chest and lungs, and improving the organs of voice and articulation, while at the same time it affords you an opportunity of directly cultivating the mind itself." (p. 42.)

The patient objects that his business is such as to leave little leisure for either exercise or amusement: the physician combats the objection, on the principle that "a man can always find some time for what he is fond of," and insists on the possibility, nay certainty, of his being able to take advantage of "some one or more of the exercises and recreations enumerated." "If, however," he adds, "you are unfortunately so circumstanced that you cannot or will not put into practice any of the measures which I have mentioned, there is yet another method to which you may have recourse, and from which I can promise you very considerable benefit; a method, too, which is perfectly in your own power, and which does not involve any sacrifice of time or any expense." (p. 44.) This method is the due graduation of the ingesta to the powers of the stomach and the wants of the system: in other words, proper attention to diet.

"As you find it impracticable to take those measures which are necessary to render your stomach equal to the work which it has to do, give it less work; do with it as you do with a weak horse: when you cannot strengthen the horse so as to render him equal to the work, you diminish the work so as to render it equal to the strength of the horse: do the same with your stomach. . . . You cannot take the stomach of another person as a measure for your own. . . . One man will be filled, even to repletion, by a quantity of food scarcely sufficient to satisfy the cravings of another man's hunger. There is no rule so good, or so general in its application, as the feeling of the stomach itself: if, after a meal, you are light and cheerful, and without flatulence or acidity, you have not eaten too much; if, on the contrary, you are oppressed or flatulent, you have erred either in the quantity or the quality of the food taken. . . . You inform me that circumstances render it difficult for you to use the means necessary to strengthen the stomach: the conclusion is obvious, you must diminish the quantity of the food. By so doing you will relieve yourself from the bilious symptoms; you will be no longer troubled with flatulence, acidity, and oppression after meals; your tongue will become clean, your spirits light, and your stomach, being no longer required to do more than it is able to do, will gradually improve in tone and temper." (pp. 45, 46.)

It is, however, only the combination of these two, by the simultaneous employment of proper exercise and proper diet, that the rational and certain cure of the disease is to be effected. Either will fail singly; but the effect of both will be triumphant.

"But this method [that is, taking only as much food as the stomach can digest], although calculated to cure your bilious symptoms, is still an imperfect method; because your sedentary, anxious, careful life (the original cause of the disorder in your stomach) will still exert its injurious influence, weakening and emaciating your muscles, shattering your nerves, and unfitting your stomach for the digestion of more than the smallest quantity of the plainest food. You will be improved indeed, because your stomach will be able to digest the diminished quantity of food; but this quantity of food being too small to impart full strength and vigour to your frame, you will still be an invalid, although no longer bilious. If you wish your cure to be complete, combine the two methods judiciously together. While your stomach continues weak, give it less work; but in the mean time do not neglect the means necessary to restore its strength. As it grows stronger, it will not only be able to do more work, but its work will be better done: it will digest its food better, and the food, digested better, will produce stronger muscle, bone, and sinew, by means of which you will be enabled to take an increased quantity of exercise without fatigue; the increased quantity of exercise will produce increased strength of stomach, and the increased strength of stomach increased strength of muscle, bone, and sinew; and so the improvement will go on in a circle: your bilious symptoms will disappear as if they were charmed away; you will lose the fastidiousness of palate and capriciousness of appetite, which a disordered stomach always generates; and you will restore to your dietary, with safety and even with advantage, various articles of food which are at present excluded from it; the bloom of health will adorn your cheeks, and vigour of body will accompany and promote vigour of mind and serenity of temper." (pp. 46, 47.)

The patient, at length thoroughly convinced by the physician's arguments, takes his leave, promising to follow out in practice the principles laid down. This he does rigidly, and on his return, *after three months*, he announces his cure to be complete. He says, "I am a new man: you have been the means, under Providence, I will not say of saving my life, but (which is much more) of enabling me to enjoy it. The existence that was almost a burthen to me has become delightful, my mind and body are both at ease, and I am able to employ their energies for the advantage of my family and the good of my fellow-creatures." (p. 49.)

All this is excellent; and we venture to say that that physician will alone treat successfully the vast class of chronic disease included under Dr. Henry's comprehensive title, who adopts and follows up the general principles of the practice recommended by him. We have been long convinced that the attempt to cure habitual biliousness or chronic indigestion by mere medicinal prescription is at least as hopeless as that of the homœopathists to cure acute diseases by their imaginary medicines; and we know of no more crying or perilous abuse in the practice of physic than the universal system of purgation followed by the routine practitioners of this country. Well may Dr. Henry call the British nation "the most purging nation in the world;" and we confess we should be proud to aid him in his laudable attempt to do away this reproach.

In admitting all this, we are by no means disposed to bestow unqualified approbation on Dr. Henry's system as here exposed: it is too narrow and exclusive; it contains some serious errors, both negative and positive, of omission and commission. He is decidedly too outrageous against the use of purgatives: his horror of them almost exceeds that of *Chanticleer* in Chaucer's Nun's Priest's Tale:

"I say furthermore
That I ne tell of laxatives no store,
For they be venomous, I wot it well:
I them defy;—I love them never a deal."

Now it is one thing to prescribe the habitual use of a remedy and to trust to it almost exclusively for the cure of a disease, and another thing to employ this remedy occasionally and under particular circumstances. So far from the use of an occasional purge being injurious in bilious disorders, it is the very reverse; and its employment in this manner does not in any way establish a necessity for its frequent repetition. Again, so far from proper aperients, even a course of them, being injurious in commencing the treatment of a bilious disorder of long standing, we hold them to be, in a majority of cases, essentially necessary, and necessary even to ensure the success of the rational anti-medicinal system advocated by Dr. Henry. The regulated diet, the air, the exercise, will evince their beneficial operation much more speedily and decidedly after the intestinal canal has been freed from old accumulations, the torpid mucous membrane has been stimulated, the glandular emunctories unloaded and excited, the plethora of the portal system relieved: nay, we are convinced that, without these preliminary measures, the hygienic treatment will entirely fail in a certain class of cases. We wish it, however, to be distinctly understood that we regard this medicinal course as merely preliminary and temporary: for permanent benefit—for the cure, properly so called,—we rely entirely, with Dr. Henry, on the rigid enforcement of hygienic measures. And here we think our author has taken too confined and exclusive a view of such measures, which has arisen from his regarding the morbid condition to be remedied as simpler than it really is. In cases of bilious disorder of long standing, almost the whole frame is implicated in all its organs and functions, in its solids and its fluids; and, in contemplating this wide and multifarious disorder, it seems, at first sight, not very likely that any one measure, however comprehensive in its operation, should be able to remedy so general an evil, but that the proper treatment should include as wide a range of agencies as can be effectively and safely applied. With this view, in addition to the air, exercise, and diet of Dr. Henry, we would enforce the use of other auxiliary means calculated to improve the disordered functions, not even excluding therefrom mild alterative aperients: among these means we would lay particular stress on bathing, warm, tepid, or cold, general or local, according to the circumstances of the case; friction to the surface; rigid attention to the state of the mind; and such *medicinal* treatment as the aggravated affections of particular organs, which almost invariably ensue in protracted cases, seem to require. It would surely be absurd to trust to exercise and diet alone for the cure of an incipient gastritis, or congested liver, or overloaded colon, or turgid abdominal veins, when we had it in our power more speedily and effectually to relieve these by the employment of leeches to the præcordia or anus; by the use of mild but effective purgations or injections; by a course of warm bathing; or even by a short course of gentle mercurials. We recommend these and other similar views to the consideration of Dr. Henry, and trust that he will not overlook their importance in preparing the next edition of his Dialogue for the press.

